# SPECT/CT-Guided Surgical Removal of a Positive External Iliac Sentinel Node in Primary Umbilical Melanoma: Report of a Case, and Up-to-Date Review of the Literature

**DOI:** 10.3389/fonc.2021.772771

**Published:** 2022-01-17

**Authors:** Franco Picciotto, Adriana Lesca, Luca Mastorino, Elena Califaretti, Luca Conti, Pietro Quaglino, Simone Ribero, Virginia Caliendo, Désirée Deandreis

**Affiliations:** ^1^ Dermatologic Surgery Section, Department of Surgery, Azienda Ospedaliera Universitaria (AOU) Città della Salute e della Scienza, Turin, Italy; ^2^ Division of Nuclear Medicine, Medical Sciences Department, University of Turin, Azienda Ospedaliera Universitaria (AOU) Città della Salute e della Scienza, Turin, Italy; ^3^ Dermatology Clinic, Medical Sciences Department, University of Turin, Turin, Italy; ^4^ Surgical Pathology Section, Oncology Department, University of Turin, Azienda Ospedaliera Universitaria (AOU) Città della Salute e della Scienza, Turin, Italy

**Keywords:** umbilical melanoma, navel melanoma, sentinel node, lymphoscintigraphy, sentinel node biopsy, SPECT/CT, thick melanoma

## Abstract

Primary umbilical melanoma is rare tumor, representing about 5% of all umbilical malignancies.The lymphatic drainage from the tumor is challenging and can be to inguinal, axillary and retroperitoneal nodes. Dynamic and static lymphoscintigraphy with single-photon emission tomography/computed tomography (SPECT/CT) and sentinel lymph node biopsy (SLNB) is a widely validated technique in patients with clinically localized melanoma to search for and quantify nodal spread of cutaneous melanoma. Moreover, it offers the surgeon the preoperative information about the number and location of the sentinel lymph nodes (SLNs), which makes SLNB easier and quicker. This is the first report of an ulcerated thick melanoma of the umbilicus metastasizing only to an external iliac lymph-node without involvement of superficial inguinal SLNs. The preoperative high-resolution ultrasound (HR-US) examination of the regional lymph node field had been normal. This case-report shows how addition of SPECT/CT to planar imaging in a patient with clinically localized umbilical melanoma can help avoid incomplete SLNB when a deep SLN is not removed. A literature review of umbilical melanoma is also provided.

## Introduction

The umbilicus is a unique anatomical site with complex vascular embryonic remnants. It is a depressed scar surrounded by a natural skinfold that measures 1.5-2 cm in diameter and lies anatomically in the middle of the abdomen (1). The umbilicus is a weak point in the abdominal wall and is vulnerable to hernia formation (2).

Neoplasms are rare in the umbilicus and can be either (Sister Mary Joseph nodule) or benign. Primary umbilical melanoma is rare, representing about 5% of all umbilical malignances (3). Excision of the entire umbilicus, including its attachment to the peritoneum, is recommended. In some cases, reconstruction of the navel region can be performed. Sentinel lymph node biopsy (SLNB) is performed for staging and to improve survival in node positive patients with intermediate thickness lesions but also for prognostic evaluation in patients with thick melanoma (i.e.≥ 4 mm) (4–6). A negative SLNB is associated with prolonged recurrence-free survival, disease-specific survival, and overall survival compared to patients with a positive SLNB (4). We herein report a case of thick primary umbilical melanoma.

The aim of this paper is to emphasize the key role of single-photon emission computed tomography with computed tomography (SPECT-CT) in identifying all sentinel lymph nodes, enabling proper staging in the age of effective adjuvant systemic therapy. The literature about this rare location of melanoma is reviewed.

## Case Report

**Figure 1:**
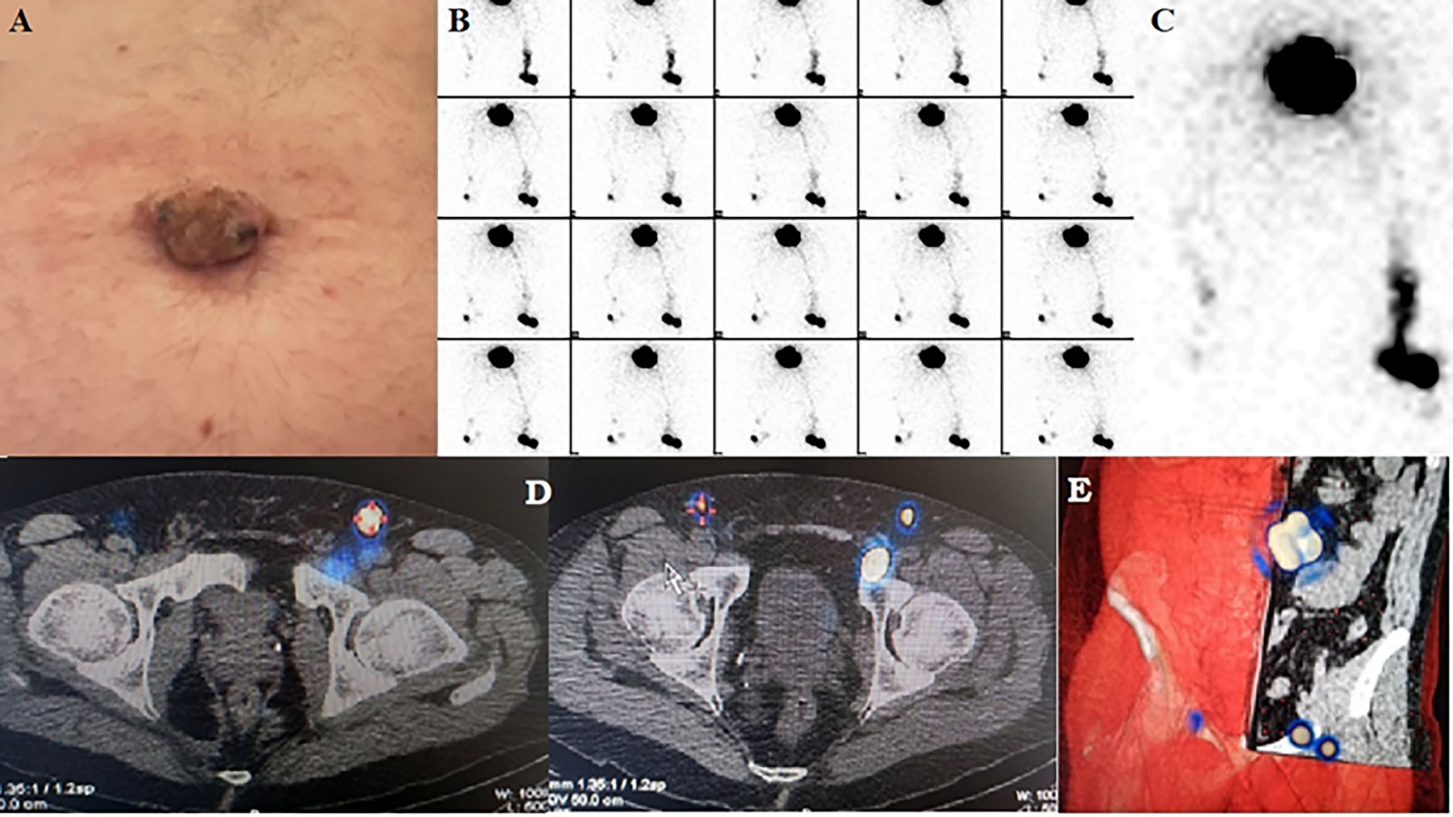
**(A)** Umbilical melanoma **(B)** Dynamic sequential images showing three lymphatic collectors departing from peritumoral injection sites **(C)** Detail showing direct drainage from the injection site to the left external iliac lymph node. **(D)** Fused axial SPECT-CT sections differentiate inguinal sentinel nodes from an external iliac sentinel node on the left. **(E)** Volume rendering SPECT-CT of the true sentinel nodes used for guiding surgery.

A 45-year-old man presented to our hospital with an umbilical skin tumor (**Figure 1A**) that had been present for about seven years and that increased in size with occasional bleeding in the last 12 months. The patient signed an informed consent for the procedures performed as well as for the use of clinical data for research. Due to the Sars-Cov2 pandemic, the patient had delayed the dermatology consultation despite an event of traumatic bleeding. No lymph node swelling was palpable on either side the inguinal regions and in the axillae. The patient underwent an excisional biopsy revealing the tumor to be melanoma. The Breslow depth was 11 mm, Clark IV with a mitotic rate of 9 per mm2. Ulceration and microscopic satellites were present. Immunostaining to S-100 protein, HMB-45, and Melan-A were all positive. The AJCC classification was pT4bN1c Total body computed tomography (CT) and 18F-FDG positron emission tomography with computed tomography (PET/CT) showed no metastases and no pathological regional lymph nodes were found by means of preoperative high resolution ultrasound (HR-US). The patient underwent wide local re-excision of the primary tumor site and SLNB. Lymphoscintigraphic mapping was performed the day before the surgery, using a double head gamma camera equipped with a CT (Discovery NM/CT 670, GE Company Milwaukee, Wisconsin, USA). A dose of 25.9 MBq of ^99m^Tc labeled Nanocolloid (Nanotop – Rotop Pharmaka GmbH, Germany) equally subdivided in four syringes, in a volume of 0.10 ml, was intradermally administered around the excisional biopsy site. Dynamic imaging (1 minute per frame in a matrix of 128x128 for 30 minutes) was started, showing bilateral lymphatic drainage to the inguinal region. Five-minute static acquisition was performed following the dynamic phase and delayed static images were then acquired in a matrix of 256x256. Subsequently, planar imaging of the thorax was carried out and SPECT/CT of the sub-umbilical region. Five-minute early static acquisition was performed with 256x256 matrix over inguinal and thorax regions, showing no axillary lymphatic drainage. Delayed static images after two hours were acquired with 256x256 matrix for five minutes over the pelvic region. The study was completed by SPECT/CT of the sub-umbilical region (step-and-shoot mode, 25 s/3°, in a 128x128 matrix size).

Summed dynamic phase images (**Figure 1B**) showed an intense focus in the left inguinal area that was considered a SLN; however, another focus with its direct lymphatic pathway gradually appeared medially to this one. Because of its direct and separate drainage from the injection site, it was deemed another SLN (**Figure 1C**). In the right inguinal area, another hot focus was quickly visualized, and classified as a contralateral right SLN. Furthermore, a faint focus appeared medially to the previous one, connected with it by a lymphatic collector, and it was deemed as a second-echelon node. SPECT-CT confirmed the presence of three SLNs in the inguinal area, one on the right side and two on the left. However, the second SLN in the left groin was found to be in the iliac area, adjacent to the inguinal canal, retropubic, near the anteromedial margin of the acetabulum. It had a normal appearance on CT images (**Figures 1D, E**). Also, the second-echelon node, detected medially on the right side, was found to be an external iliac node. No SLNs were seen on planar imaging of the thorax. The SLNs were marked on the skin in orthogonal projections.

Using general anesthesia, all three SLNs were identified after intradermal injection of vital blue dye and with intra-operative use of a hand-held gamma ray detection probe. All three sentinel nodes were stained blue and were removed.

**Figure 2:**
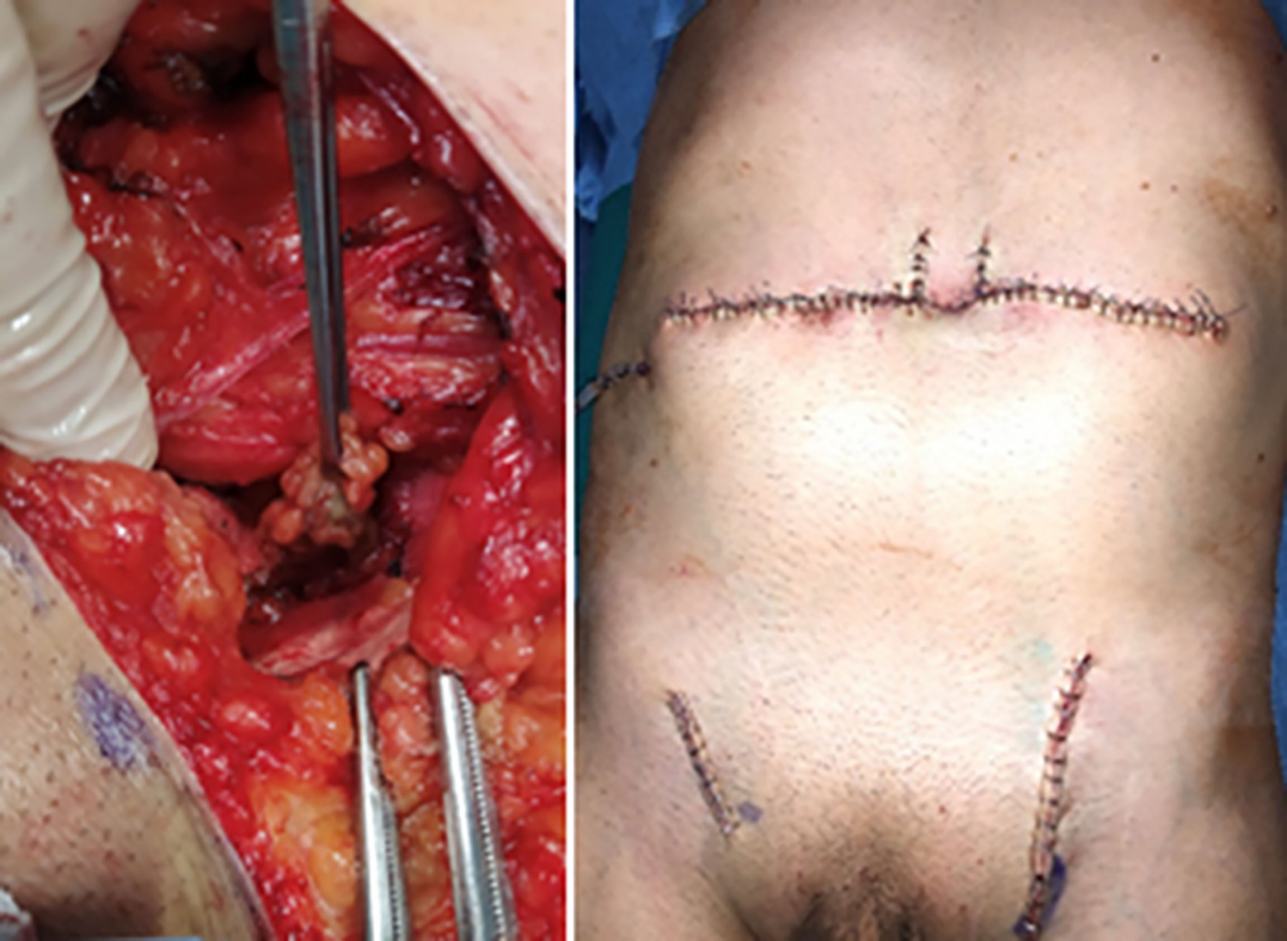
Operative views: Sentinel node in the left external iliac area and postoperative photograph.

The primary tumor was excised including the umbilicus, with >2-cm lateral margins, and down to the underlying peritoneum (**Figure 2**). At histopathological analysis, the two superficial bilateral inguinal SLNs were negative. The deep one taken from the left iliac site was positive for metastasis, with a size of 1.6 x 1.2 x 0.6 cm and a tumor burden of 1.5 mm as maximum diameter of largest deposit. This allowed a more precise pathological staging as **pT4bN2C** and an adequate stratification of risk, based on the coexistence of ulceration along with high sentinel nodal tumor burden (i.e. >1.0 mm) (7).

Inguino/iliac/obturator lymph node dissection was not performed, since active surveillance has been demonstrated to have equivalent survival outcomes to completion lymphadenectomy (8). Given positive result for BRAF V600E mutation, the patient was started on adjuvant combination therapy with BRAF and MEK inhibitors along with focused HR-US surveillance of nodal basins. At follow-up, 4 months after surgery, HR-US of nodal basins (axillary and inguinal), brain MRI with contrast and Whole Body 18F-FDG PET/CT were all normal.

## Discussion

Primary cutaneous malignant melanoma involving the umbilicus is a rare entity and, to our knowledge, only 32 cases have been described in the literature since 1975 (9–24). Given the anatomical site of the umbilicus, lymphatic drainage and thus dissemination, may occur to the inguinal area and to the axilla (25), but also to parailiac lymph nodes, even without involvement of the superficial inguinal nodes.

Although the data were incomplete in most publications, the literature review showed some specific features of navel melanoma. First, there was a marked female prevalence (24 females and 8 males) (**Table 1**). It has been suggested that the presence of terminal body hairs in male patients could represent a natural UV-protective barrier (9). The median age of diagnosis was 58 years, with a great range of 28-84 years. The majority of melanomas were of the nodular type with a vertical growth phase. There were three cases of *in situ* melanoma. The median Breslow thickness was 2.5 mm with a range of 0.42-11, reflecting that navel melanoma is often detected at a fairly advanced stage, suggesting that melanoma arising in this body site is more aggressive than elsewhere.

**Table 1:** Summary of literature findings on umbilical melanoma.

Author	Sex/Age	Type	Breslow (mm)	Clark	Time from occurrence	Pre-existing nevus	SLNB	Therapy	Relapse	Survival	BRAF
Ki Wei Tan (9) 2021	F/59	SSM	6.4	IV	2 weeks	Yes	Yes	Adjuvant Nivolumab	Nodal Lung - Bone	NA	Wild-type
Kovitwanichkanont (11) 2020	F/74	Nodular amelanotic ulcerated	21	IV	Long standing		Yes	Adjuvant Nivolumab	4 months loco-regional	Alive	Wild-type
Kovitwanichkanont (11) 2020	F/44	Ulcerated SSM	2.2	IV	Several years		NA	No therapy		3 years, alive	
Charles (10) 2020 (7 cases)	6 F, 1 M/52 years (39-72)	6 SSM, 1 NM	4.21 (0.65-15.6)			5 (4 at histology)	Yes	1 adjuvant target therapy then Ipilimumab, one clinical trial	1 liver, 1 disseminated	2 deaths at 10 and 17 months	2 wild-type, 2 mutant
Costa-Silva (12) 2017	F/81	Ulcerated	5.6								
Suzuki (13) 2016	F/83		11	IV	2 weeks	No	Yes	No		15 months, alive	
Di Monta (14) 2015	F/33	NM ulcerated	4.0				Yes	Anti-MEK/anti-BRAF/Ipilimumab	6 months - Local then diffuse	28 months, died	Mutant
Di Monta (14) 2015	F/50	SSM ulcerated	2.5				Yes			21 months, alive	
Di Monta (14) 2015	M/77		3						12 months, inguinal nodes, then bone/liver mets	8 months, died	
Song (15) 2013	M/62		3	IV	1 month	Reported nevus 4 years before				36 months, alive	
Navysany (16) 2013	F/NA		4.1							3 months, alive	
Dessy (17)2013	F/36	In situ				Present at histology				3 months, alive	
Papalas (3) 2011	M/41	NM	1.75	IV							
Papalas (3) 2011	F/28	In situ		I							
Papalas (3) 2011	F/58	SSM	1.87	IV							
Papalas (3) 2011	F/84	SSM	4.16	IV							
Papalas (3) 2011	M/35	In situ		I							
Papalas (3) 2011	F/47	SSM	0.42	II							
Zaccagna (18) 2011	F/60	VGP	2.8	IV			Yes			86 months, alive	
Cecchi (19) 2009	F/77	SSM-VGP ulcerated, achromic	2.3	IV	4 years		Yes			1 year, alive	
Mangas (20) 2008	M/63		0.8	III	Unknown	Present at histology					
Campos-Munoz (21) 2007	F/34	SSM	1.06	III	1 month	Present at histology	Yes			18 months, alive	
Meine (22) 2003	F/69	SSM -VGP	1.88	IV	2 months				Local relapse	3 year, alive	
Colonna (23) 1999	F/58	NM plus satellites		V		Reported nevus		Dacarbazine	9 months, visceral	10 months, died	
Colonna (23) 1999	M/30			V	18 months	Reported nevus			Visceral	30 days, died	
Hashiro (24) 1998	M/58			III	Reported since childhood	Present at histology		Poly-chemotherapy		15 months, alive	

SLN, Sentinel Node Biopsy; NM, Nodular Melanoma; SSM, Superficial Spreading Melanoma; VGP, Vertical-Growth-Phase; NA, Not Available.

Supporting this hypothesis is the finding that six of the seventeen patients for whom disease follow-up data were available have developed a disease relapse and died of melanoma after a time interval of 30 days to 28 months from the initial operation. SLNB is a crucial element in the management of melanoma patients (26, 27). It provides prognostic and staging information, improves regional disease control in cases where complete lymphadenectomy is performed, improves survival of node-positive patients in case of an intermediate Breslow thickness melanoma and helps selecting patients who may benefit from adjuvant systemic treatment (27). Only eight of the articles in our review reported performance of SLNB in a total of fourteen patients with primary umbilical melanoma, with nodal positivity in only four patients.

The main site of metastasis was the inguinal node field in all four patients, with a total of five positive lymph nodes. One of these patients also had a positive SLN in the left axilla (**Table 2**).

**Table 2:** Synopsis of Sentinel Node Biopsy Results.

Author	SLNB Result	Draining Node Field
Ki Wei Tan (9) 2021	Positive	Right Inguinal – Left Inguinal
Kovitwanichkanont (11) 2020 (Case 1)	Positive	Left Inguinal
Kovitwanichkanont (11) 2020 (Case 2)	Negative	NA
Charles (10) 2020 (Case 1)	Positive	Left Inguinal – Left Axilla
Charles (10) 2020 (Case 2)	Positive	Right Inguinal – Left Inguinal
Charles (10) 2020 (Case 3)	Negative	Right Inguinal – Left Inguinal
Charles (10) 2020 (Case 4)	Negative	Right Inguinal – Left Inguinal
Charles (10) 2020 (Case 5)	Negative	Right Inguinal – Left Inguinal
Charles (10) 2020 (Case 6)	Failed Test	Failure of Tracer Migration
Suzuki (13) 2016	Negative	Right Inguinal – Left Inguinal
Di Monta (14) 2015 (Case 1)	Negative	Left Inguinal – Left Axilla – Right Axilla
Di Monta (14) 2015 (Case 2)	Negative	Right Inguinal
Zaccagna (18) 2011	Negative	NA
Cecchi (19) 2009	Negative	Right Inguinal – Left Inguinal
Campos-Munoz (21) 2007	Negative	Left Axilla
**Total**	**15**	

NA, Not Available.

It is essential to use the correct definition of a SLN and to select an accurate technique for its retrieval. Most experts define a sentinel node as any lymph node on a direct lymphatic drainage pathway and most also use planar lymphoscintigraphy starting with dynamic imaging to delineate the lymph vessels. This is followed by delayed static imaging and SPECT/CT. SPECT/CT superimposes the anatomical information of CT onto the functional images of lymphoscintigraphy to reveal the precise 3D anatomical location of SLN(s) (28, 29).

The images are displayed as tomographic sections in the transaxial, coronal, sagittal planes and can also be displayed as 3D volume rendering images. It can provide additional information, especially when the tumor is located in the head, neck, or torso (28) and helps to localize pelvic SLNs (29). Moreover, the use of SPECT/CT is associated with a higher SLN excision rate and lower local relapse rate (30).

In brief, SPECT/CT defines with greater accuracy the anatomical position of SLN(s), clarifies unusual drainage pathways, and identifies pathologically enlarged lymph nodes (31). At our institution, SPECT/CT is performed routinely in addition to planar imaging. In the subject of this case report the role of the SPECT/CT was decisive, allowing the surgeon to be guided toward an external iliac positive sentinel lymph node, located below the fascia of the external oblique muscle.

In the present case, removal of the only iliac metastatic node led to a more precise classification (pT4bN2C), without changing the stage (III C).

This is furthermore the first reported case of a lymph node metastasis spread only in an external iliac node, without involvement of the superficial inguinal sentinel nodes. Now that adjuvant systemic therapy is becoming common in patients with even minimal nodal involvement, SLNB is becoming even more important.

## Conclusion

In the new era of effective adjuvant therapy, accurate staging of patients with melanoma has become even more important than it was before. The current case report demonstrates how accurate staging can be accomplished in a patient with a melanoma in the umbilicus, a rare location at the crossroads of lymphatic drainage pathways. Dynamic lymphoscintigraphy indicated the SLNs and SPECT/CT showed the node that eventually proved to be the only metastatic one in its unusual anatomic habitat in the pelvis.

## Data Availability Statement

The original contributions presented in the study are included in the article/supplementary material. Further inquiries can be directed to the corresponding author.

## Ethics Statement

Ethical approval was not provided for this study on human participants because this is a case report and a review of the literature. The patient signed an informed consent for the use of own data. The patients/participants provided their written informed consent to participate in this study. Written informed consent was obtained from the individual(s) for the publication of any potentially identifiable images or data included in this article.

## Author Contributions

DD, AL, and EC took care of scintigraphic investigation. FP and VC conducted the surgery. LC carried out the histopathological study. PQ, SR, and LM revised the literature. All authors contributed to the article and approved the submitted version.

## Conflict of Interest

The authors declare that the research was conducted in the absence of any commercial or financial relationships that could be construed as a potential conflict of interest.

## Publisher’s Note

All claims expressed in this article are solely those of the authors and do not necessarily represent those of their affiliated organizations, or those of the publisher, the editors and the reviewers. Any product that may be evaluated in this article, or claim that may be made by its manufacturer, is not guaranteed or endorsed by the publisher.
